# Paraplegia in a chiropractic patient secondary to atraumatic dural arteriovenous fistula with perimedullary hypertension: case report

**DOI:** 10.1186/2045-709X-21-23

**Published:** 2013-07-08

**Authors:** Stephen M Foreman, Michael J Stahl, Gary D Schultz

**Affiliations:** 1 Private practice of chiropractic, West Hills, California, USA; 2 University of Western States, Portland, Oregon, USA

**Keywords:** Chiropractic, Manipulation, Dural arteriovenous fistula, Paraplegia, Pontomesencephalic venous congestion, Complication, Stroke

## Abstract

Intracranial dural arteriovenous fistulas are abnormal communications between higher-pressure arterial circulation and lower-pressure venous circulation. This abnormal communication can result in important and frequently misdiagnosed neurological abnormalities.

A case of rapid onset paraplegia following cervical chiropractic manipulation is reviewed. The patient’s generalized spinal cord edema, lower extremity paraplegia and upper extremity weakness, were initially believed to be a complication of the cervical spinal manipulation that had occurred earlier on the day of admission. Subsequent diagnostic testing determined the patient suffered from impaired circulation of the cervical spinal cord produced by a Type V intracranial arteriovenous fistula and resultant venous hypertension in the pontomesencephalic and anterior spinal veins.

The clinical and imaging findings of an intracranial dural arteriovenous fistula with pontomesencephalic venous congestion and paraplegia are reviewed.

This case report emphasizes the importance of thorough and serial diagnostic imaging in the presence of sudden onset paraplegia and the potential for error when concluding atypical neurological presentations are the result of therapeutic misadventure.

## Background

Dural arteriovenous fistulas (DAVF) are abnormal communications between arterial and venous circulation that may arise either spontaneously or as a result of trauma [1]. DAVFs account for 15% of all cerebrovascular malformations [2]. Previous studies have determined DAVFs occur in both sexes equally and the onset of symptoms most commonly occurs between the third and sixth decade of life [3]. The frequent association of patients with DAVF and cerebral venous sinus thrombosis [4] indicates spontaneous DAVFs may be the result of venous thrombosis and venous hypertension [5]. The symptoms and physical findings of DAVF vary greatly depending on the affected anatomy and resulting vascular changes, flow restrictions within the DAVF, the presence and size of arterial feeders and the location and direction of venous drainage. Symptoms, produced by the shunting of higher-pressure arterial flow into the lower-pressure venous system appear to depend on the direction of venous drainage [6] and this in turn determines whether the symptoms will be benign or aggressive.

Cougard [7] reviewed 258 cases of cerebral DAVFs and classified the patients into groups with aggressive and nonaggressive neurological symptoms. Aggressive neurological symptoms included “intracranial hypertension (i.e., headache, nausea or vomiting, transient visual defect or visual loss and papilledema), intracranial hemorrhage, focal deficits, seizures, altered mental status and ascending myelopathy.” The nonaggressive neurological symptoms included isolated headache, bruit and all ocular symptoms not related to intracranial myelopathy.

Cougard’s [7] classification of DAVF presentation, based on the drainage pattern (Table 1), helps explain the variability of clinical presentation. Cougard classified the pattern of DAVF drainage into five types, with the associated neurological symptoms progressively worsening from Type I to Type V.

**Table 1:** Classifications of intracranial dural arteriovenous fistulas

**Type**	**Description**
Type I	Drainage into a sinus without flow restriction
Type II	Drainage into a sinus with flow restriction and reflux into other sinuses or into cortical veins
Type III	Drainage into a cortical vein
Type IV	Drainage into a cortical vein with a large varix
Type V	Spinous perimedullary venous drainage

Adapted from: [7].

Type I, the most common and least aggressive form of DAVF, is based on arterial drainage into an intracranial sinus without flow restriction. Type I DAVFs are generally benign and may be asymptomatic [8]. In Type II, reflux produces intracranial hypertension in 20% of cases and hemorrhage in 10%. Intracranial hemorrhage is present in 40% of the Type III cases and in 65% of Type IV. Finally, Type V cases, with spinal perimedullary drainage, produce an ascending myelopathy in 50% of cases. The development of ascending myelopathy from perimedullary venous hypertension, first documented in 1982 [9], is rare and was only present in 2.3% (n=6) of Cougard’s 258 cases [7]. The Type V pattern of drainage is the least common and produces the most aggressive and serious neurological symptoms. The ascending myelopathy associated with Type V cases may result in brainstem dysfunction manifesting as respiratory insufficiency [1]. Brainstem involvement seems to be related to involvement of the anterior pontomesencephalic and petrosal vein [10].

## Case presentation

**Figure 1:**
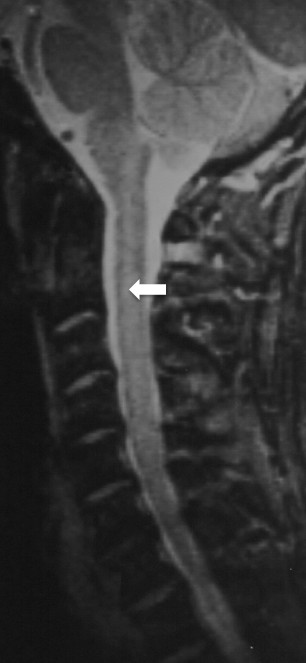
**Initial Sagittal MR scan of cervical spine.** MR scan, T2 weighted, sagittal view without contrast, reveals diffuse increased signal intensity (arrow) covering the entirety of the medulla and extending to C5-6. The high signal intensity is affecting the central portion of the spinal cord and sparing the outer (subpial) tissues. Initial review concluded the findings were consistent with “infarction vs. contusion.”

A 59-year-old male presented to a chiropractic office with a three-week history of pain in the lumbar and lower cervical spine, which began subsequent to moving boxes in his home. Physical examination findings were consistent with musculoskeletal strain and the chiropractor performed spinal manipulation in both the cervical and lumbar spine without consequence. Later that same day the patient began to experience muscular weakness in his lower extremities and loss of bladder control. These serious neurological symptoms prompted him to seek care within an emergency room. MR scans of the cervical spine and brain, with and without contrast, were performed and initially interpreted as normal. A secondary review of the cervical spine MR noted swelling of the cervical cord, without hemorrhage from C1 to C6 (Figure 1), consistent with a clinical impression of “infarction vs. contusion.” Attending physicians at the ER, based on the patient’s history of cervical manipulation earlier that same day, erroneously concluded the patient suffered spinal cord contusion secondary to a manipulation with excessive force.

**Figure 2:**
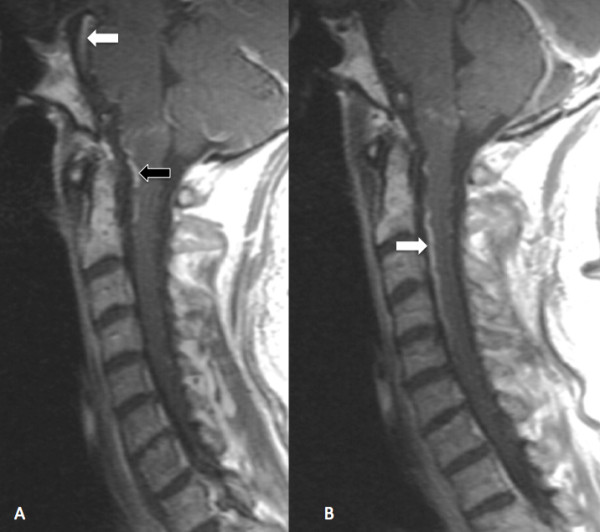
**Follow-up Sagittal MR scan of cervical spine. (A)** MR scan, T1 weighted, sagittal view without contrast, reveals increased signal intensity and size of the pontomesencephalic vein (white arrow). Increased signal intensity is also noted in the mesenecephalic vein and anterior spinal vein (black arrow), both of which are continuations of the pontomesencephalic vein. **(B)** The next image in the series provides a clearer view of the increased signal intensity in the anterior spinal vein, (white arrow) well visualized between the lower aspects of C2 to mid portion of C5.

The patient was hospitalized and his neurological status continued to deteriorate over the ensuing days. The continued neurological deterioration, along with the subsequent review of the initial MR scan, called into question the initial etiological conclusions related to the chiropractic manipulation. The widespread edematous changes seen in Figure 1, extending from the medulla to C5, were inconsistent with a theory that a single manipulation directed to a specific spinal level, could produce such findings. The edematous cord changes, ascending myelopathy and quality of the initial MR, led to acquisition of a second cervical MR (Figure 2A and B).

**Figure 3:**
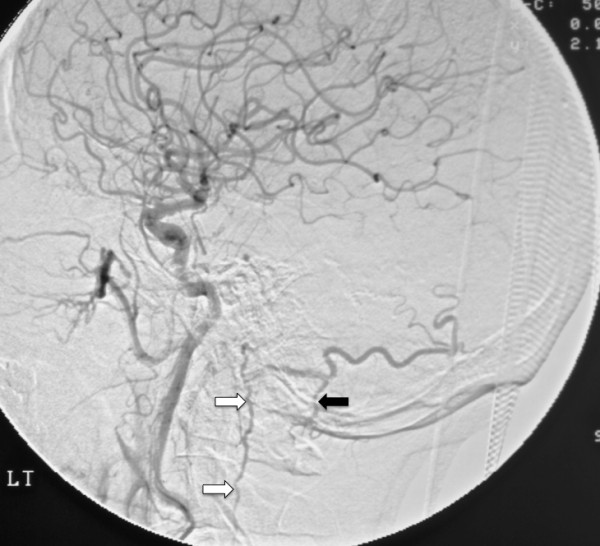
**Digital subtraction angiogram of the cerebellum.** A digital subtraction angiogram, early arterial phase, performed by injection of the left internal carotid artery. The anterior spinal vein (white arrows) was visualized during the study from C1 to C5. The posterior spinal vein (black arrow) was visualized from C1-2. No visualization of the anterior and posterior spinal vein was noted in the angiogram performed subsequent to the insertion of the neurovascular clip.

The second cervical MR scan, performed two days after admission, revealed increased signal intensity along the path of the pontomesencephic vein anterior to the medulla and the anterior spinal vein as it continues along the anterior surface of the spinal cord. These new MR findings also revealed the possible presence of an intracranial DAVF and prompted the performance of a cerebral angiogram. The cerebral angiogram revealed a Type V DAVF draining into the perimedullary venous system. The arterial phase of the angiogram (Figure 3) revealed contrast enhancement of the anterior and posterior spinal vein. Surgical inspection, via a retrosigmoid approach, revealed a fistula between the hypophysealmeningeal trunk, a branch off the internal carotid artery, and the pontomesencephalic vein. Arterialized blood within the pontomesencephalic vein was visualized during surgery. A neurovascular clip was placed over the fistula, between the arterial and venous sides and restored the normal venous color within the pontomesencephalic vein. Surgical intervention halted the neurological deterioration. Minimal function returned with rehabilitation.

## Conclusions

This case illustrates the potential difficulties in determining the etiology of rapidly developing paraplegia and ascending myelopathy. The abnormalities noted in Figure 1 were initially overlooked and only appreciated on a secondary review, when the patient’s myelopathy began to ascend. The observations on the second review of Figure 1, combined with the history of chiropractic manipulation earlier on the day of admission, led to an erroneous etiological conclusion. Attending physicians initially believed the patient had experienced a rare complication from cervical manipulation, an impression that prevailed for several days, delaying proper intervention. The location and breadth of the patient’s ischemia and the absence of similar cases in the literature, should have argued against chiropractic manipulation as the cause of the patient’s ascending myelopathy. The admitting physician’s prolonged focus on a possible complication from chiropractic manipulation may be characterized as an anchoring bias [11].

The continued deterioration of the patient’s condition over the first 48 hours after admission stimulated initiation of serial diagnostic testing, including a second MR scan of the cervical spine. Increased signal intensity within the ponto/medulo/spinal venous network on the second cervical MR examination (Figure 2) raised the specter of an intracranial etiology. Angiography identified the Type V intracranial DAVF as the true cause of the ascending myelopathy.

This case also illustrates the value of multiple as well as serial advanced diagnostic imaging procedures capable of demonstrating pathology responsible for such clinical presentations. Serial studies are warranted (even when the time elapsed between studies is small) when clinical deterioration of the patient’s status suggests that significant changes in the pathoanatomy have occurred. This case also illustrates how complex problems are sometimes inadequately evaluated from a single diagnostic study. The actual cause of the patient’s myelopathy was only discovered after higher quality MR scans and angiography isolated the DAVF. This case also demonstrates the potential for attending clinicians to formulate inaccurate etiological opinions of causation based solely on temporal association in patients with a history of receiving chiropractic manipulation. This case further illustrates the danger of assumption, similar to other peer-reviewed papers reporting neurological complications from chiropractic manipulation [12,13], that were later shown to be inaccurate [14,15].

## Consent

Written informed consent was obtained from the patient for publication of this case report and any accompanying images. A copy of the written consent is available for review by the Editor-in-Chief of this journal.

## Abbreviations

DAVF: Dural arteriovenous fistula; MR: Magnetic resonance.

## Competing interests

The authors declare they have no competing interests.

## Authors’ contributions

SMF conducted the initial review of the case and prepared the first draft of the manuscript. MJS and GDS participated in the conception of the report, the revision and coordination of the final manuscript. GDS obtained reference literature used in the manuscript. All authors read and approved of the final manuscript.
